# Toxicology assessment of manganese oxide nanomaterials with enhanced electrochemical properties using human in vitro models representing different exposure routes

**DOI:** 10.1038/s41598-022-25483-w

**Published:** 2022-12-05

**Authors:** Natalia Fernández-Pampín, Juan José González Plaza, Alejandra García-Gómez, Elisa Peña, Carlos Rumbo, Rocío Barros, Sonia Martel-Martín, Santiago Aparicio, Juan Antonio Tamayo-Ramos

**Affiliations:** 1 International Research Center in Critical Raw Materials-ICCRAM, Universidad de Burgos, Plaza Misael Bañuelos s/n, 09001 Burgos, Spain; 2 Gnanomat S.L., Campus Cantoblanco, Madrid Science Park, c/ Faraday 7, 28049 Madrid, Spain; 3 Department of Chemistry, Universidad de Burgos, 09001 Burgos, Spain

**Keywords:** Biological models, Nanotoxicology

## Abstract

In the present study, a comparative human toxicity assessment between newly developed Mn_3_O_4_ nanoparticles with enhanced electrochemical properties (GNA35) and their precursor material (Mn_3_O_4_) was performed, employing different in vitro cellular models representing main exposure routes (inhalation, intestinal and dermal contact), namely the human alveolar carcinoma epithelial cell line (A549), the human colorectal adenocarcinoma cell line (HT29), and the reconstructed 3D human epidermal model EpiDerm. The obtained results showed that Mn_3_O_4_ and GNA35 harbour similar morphological characteristics, whereas differences were observed in relation to their surface area and electrochemical properties. In regard to their toxicological properties, both nanomaterials induced ROS in the A549 and HT29 cell lines, while cell viability reduction was only observed in the A549 cells. Concerning their skin irritation potential, the studied nanomaterials did not cause a reduction of the skin tissue viability in the test conditions nor interleukin 1 alpha (IL- 1 α) release. Therefore, they can be considered as not irritant nanomaterials according to EU and Globally Harmonized System of Classification and Labelling Chemicals. Our findings provide new insights about the potential harmful effects of Mn_3_O_4_ nanomaterials with different properties, demonstrating that the hazard assessment using different human in vitro models is a critical aspect to increase the knowledge on their potential impact upon different exposure routes.

## Introduction

Manganese is one of the most abundant metals on the planet^1^, mainly found in nature as MnO_2_ or Mn_3_O_4_^2^, and a critical raw material in a number of applications, including the global steel industry, the production of aluminium alloys (e.g. for beverage cans), and the development of Mn batteries^3^. In the last two decades, manganese oxide nanomaterials have become relevant compounds in a variety of areas, such as the development of energy storage devices (lithium-ion batteries, capacitors), biomedical applications, catalysis, etc. due to their inherent divergence in redox properties, morphology, crystalline structure, and surface nanoarchitectures^4,5^. Specifically, Mn_3_O_4_ nanoparticles are of particular interest in the development of supercapacitators, due to their single black manganese structure at room temperature, good stability and controllable microstructure^6^. Within this particular field, efforts are being done to improve the characteristics of Mn_3_O_4_ based electrodes, e.g. to enhance the specific surface area and the conductivity of the nanomaterial^6,7^.

Besides its industrial relevance, manganese is an essential micronutrient needed for plant growth and for maintaining animals’ health and well-being. Due to its central role for the organism, Mn levels have to be kept finely regulated in order to keep homeostatic concentrations of this metal^8^. While lack of this element causes detrimental effects over the organism, excess impairs the proper functioning of the cell. One of the basal problems is the reactivity of the ion itself. Manganese can act over endogenous H_2_O_2_ and mediate the release of reactive oxygen species (ROS), such as OH through Fenton type of reactions^9^. The accumulation of ROS can lead to apoptotic events^10^, further compromising the stability of tissues and subsequently affecting the functionality of entire organs^11^. Choi et al. have shown that MnOx nanoparticles can induce ROS in human lung adenocarcinoma cells (A549)^12^. Moreover, Frick et al. have reported than Mn_3_O_4_ interacts with gluthatione (GSH) and it can induce apoptosis in rat type II epithelial cells, CCL149 cells^13^.

The increase in manganese oxide applications, particularly in nanomaterial form, may consequently lead to an increase in the human exposure and risk, due to their potential toxicity. Regarding this, it has been demonstrated that the main source of Mn intoxication is through occupational exposure. Recent studies suggest that low-levels Mn in the working environment can produce harmful effects on human health. Moreover, other factors are related to Mn toxicity such as age, gender, ethnicity, genetics, location, and pre-existing medical conditions^14^. These risks are especially evident on at the manufacturer’s site, where workers (miners, smelters, welders and workers in dry-cell battery factories) are subjected to occupational exposure. Inhalation, intestinal absorption (mainly via mucociliary clearance and swallowing of respiratory secretions) and dermal contact, are the main routes of nanomaterials exposure^15^. Therefore, there is a need to evaluate the possible associated hazards of novel nanomaterials containing manganese, considering different potential exposure routes. Results obtained from nanotoxicology assays can be an important instrument for the industry, in the development of safer formulations. In the current study, a novel engineered Mn_3_O_4_ nanomaterial, namely GNA35, and its precursor material (Mn_3_O_4_), have been comparatively assessed at physicochemical and toxicological level, employing three human in vitro models resembling the main systems of the human body and organs associated with the routes of nanomaterials exposure: respiratory system (human alveolar carcinoma epithelial cell line, A549), gastrointestinal system (human colorectal adenocarcinoma cell line, HT29) and skin (reconstructed human epidermal model EpiDerm).

## Results

### Nanomaterials physico-chemical characterization

**Figure 1:**
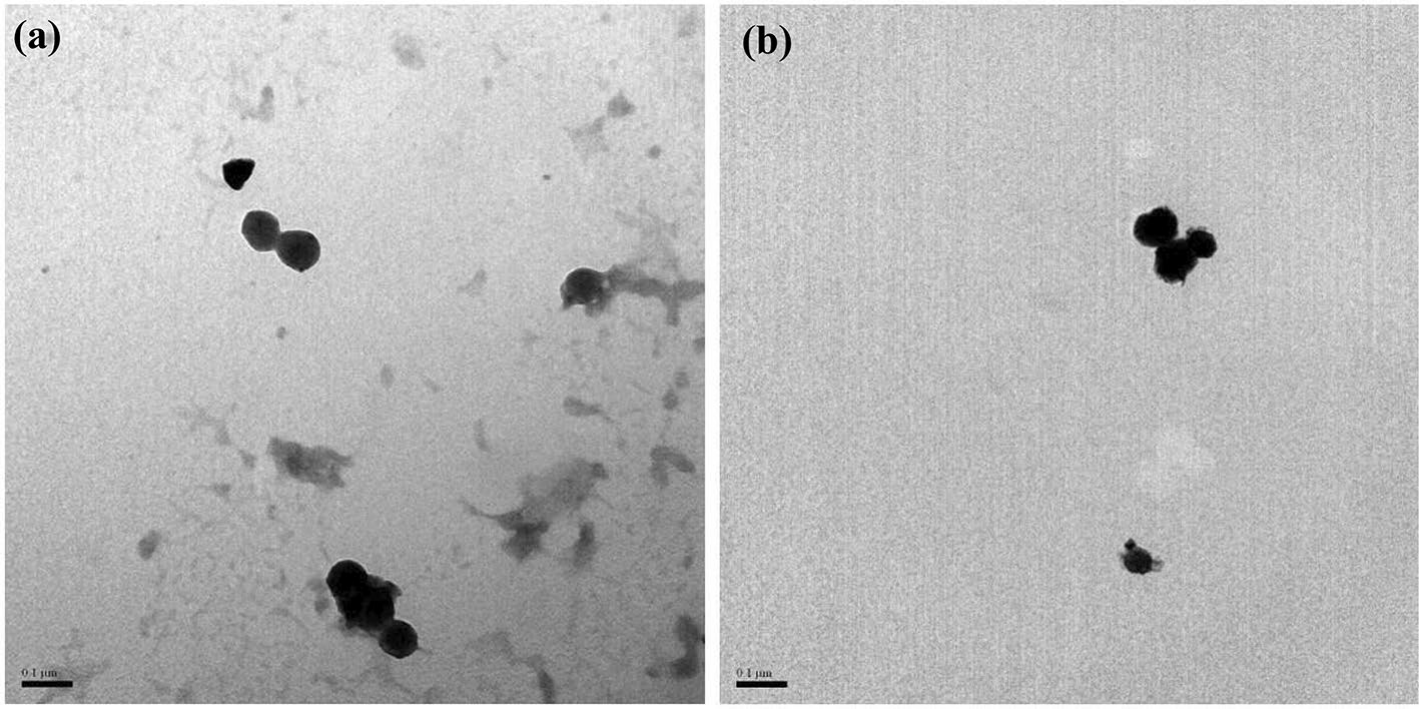
TEM analysis of Mn_3_O_4_ (**a**) and GNA35 (**b**) nanomaterials. Scale (0.1 µm) is shown on each of the panels.

GNA35 nanoparticles were synthesized aiming to obtain a nanomaterial with improved characteristics, such as surface area and conductivity^6,7^, for the development of supercapacitators. This was done using commercial Mn_3_O_4_ as precursor (named from now on “Mn_3_O_4_”), and following procedures described in Patent number ES2678419A1. Prior to their toxicological assessment, the physico-chemical properties of the precursor material (Mn_3_O_4_) and the synthesized nanomaterial (GNA35) were determined, using several methodologies to obtain insights into their morphology, size, surface charge, crystallinity, surface area and electrochemical performance. Firstly, a TEM analysis was performed to observe their morphological characteristics and size. Figure 1 shows representative images of Mn_3_O_4_ (Fig. 1a) and GNA35 (Fig. 1b), (additional TEM images can be found at Supplementary Fig. S1 and S2). As it can be observed, both the precursor and the new synthesized material showed to have round shape and a similar diameter, of around 100 nm.

Particle size and stability was determined as well through dynamic light scattering (DLS) and ζ-potential, in ultrapure water and in human cells culture media (DMEM + 1% FBS) suspensions, at a concentration of 20 mg L^−1^. The observed average values for the nanomaterials (NMs) resuspended in water were significantly higher than those observed through TEM: 347 ± 69 nm for Mn_3_O_4_ and 364 ± 91 nm for GNA35, while their respective ζ-potential was – 16.4 ± 0.9 and – 20.9 ± 0.6. When the samples were resuspended in culture media, only GNA35 was stable enough to perform DLS and ζ-potential measurements, which were very similar to those obtained in water (DLS: 338 ± 0,6 nm; ζ-potential: − 23.0 ± 3.0).

**Figure 2:**
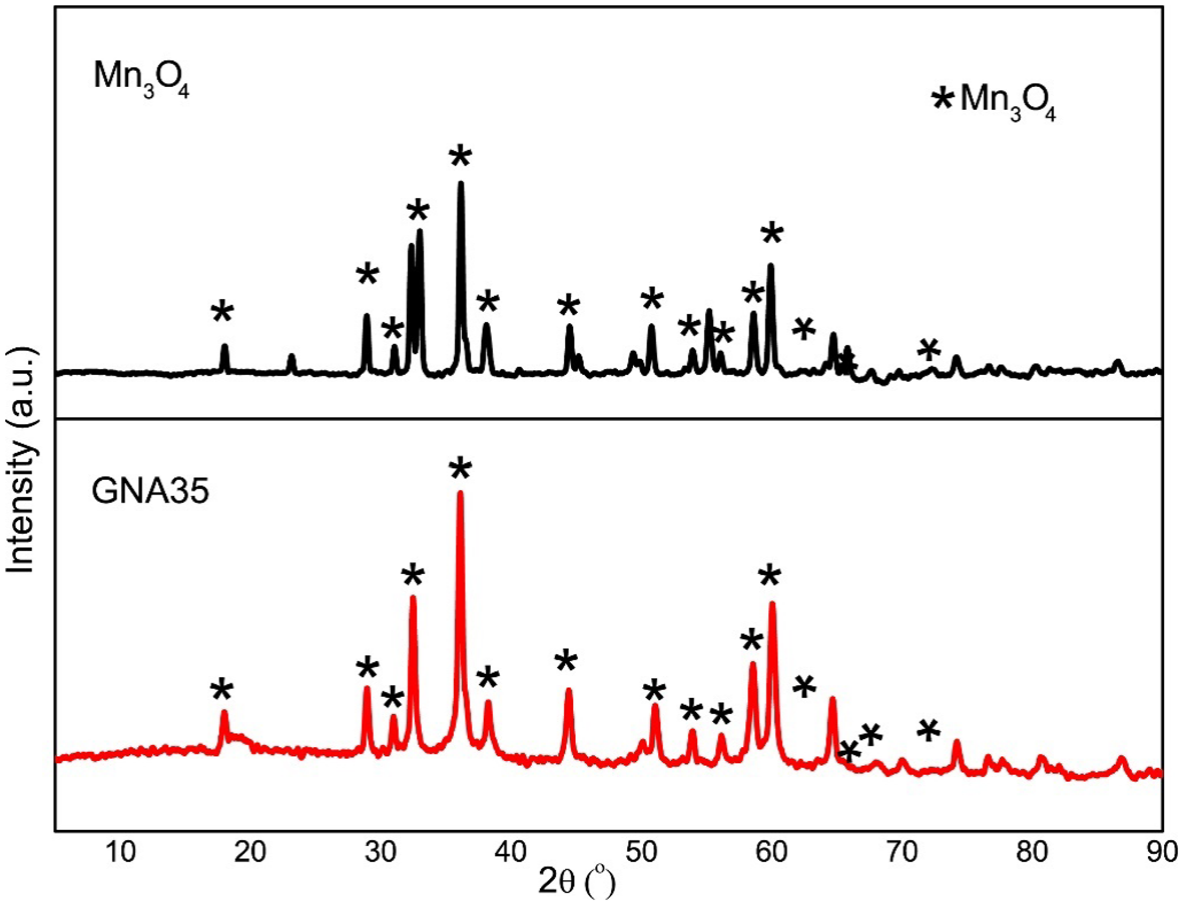
XRD spectra of Mn_3_O_4_ and GNA35 crystalline nanomaterials, with some differences in its composition. The stars refer to the peaks that correspond to the pattern showing the diffraction peaks of hausmannite Mn_3_O_4_.

The chemical composition and crystalline phase of Mn_3_O_4_ and GNA35 were determined by X-ray diffraction analysis. The assays were performed by X-Ray Polycrystalline Diffraction. The XRD analysis indicate that the diffraction spectra of Mn_3_O_4_ and GNA35 obtained are correlated with the hausmannite (Mn_3_O_4_; reference pattern 00-024-0734). However, in these spectra it can be observed a higher amount of impurities, which could be present in the raw material (Fig. 2).

Analysis of nitrogen adsorption–desorption isotherms was carried out to determine the porosity of samples. The results have demonstrated a good agreement between surface area and accumulated charge, which is key in electrode materials for energy storage devices. The estimated BET area was 14 and 39 m^2^g^−1^ for Mn_3_O_4_ and GNA35 respectively, demonstrating that the new synthesized material possesses increased specific surface, which could result in improved physical–chemical properties for charge accumulation.

**Figure 3:**
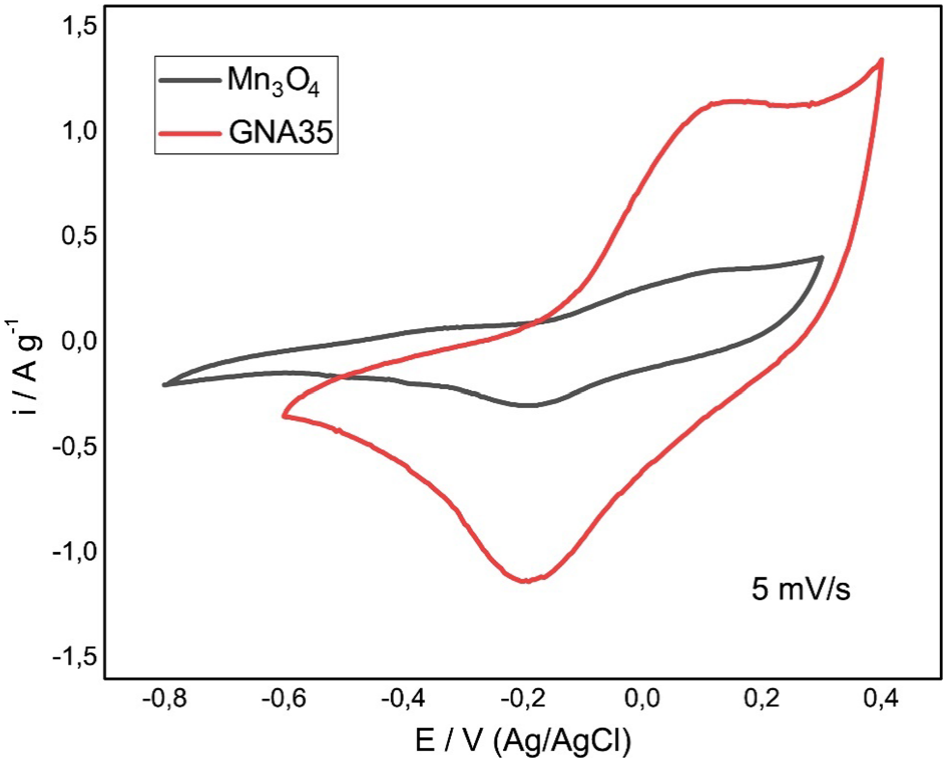
Electrochemical characterization of Mn_3_O_4_ and GNA35 electrodes: Cyclic voltammetry curves in 1 M KOH aqueous electrolyteat scan rate of 5 mV s^−1^ in a range of voltage between − 0.8 and 0.4 V (Ag/AgCl). Specific capacitance of 16 and 150 F g^−1^ were calculated at 1 A g^−1^ for Mn_3_O_4_ and GNA35, respectively.

Cyclic voltammetry was employed to determine the amount of charge that can be accumulated by Mn_3_O_4_ and GNA35. As it can be seen in Fig. 3, the wider area observed for GNA35 indicates that the accumulated charge in this nanomaterial is greater than in the raw nanomaterial. Furthermore, the specific capacitance (amount of stored charge per unit change in electric potential), calculated from galvanostatic charge/discharge measurements at 1 A g^−1^ for GNA35 was 150 F g^−1^, being 16 F g^−1^ for Mn_3_O_4_. These results corroborate that the new synthesized nanoparticles have an improved electrochemical response when compared to the starting material, ensuring a better performance as electrode in an energy storage device.

### Evaluation of GNA35 and Mn_3_O_4_ toxicity in the human alveolar carcinoma epithelial cells A549

**Figure 4:**
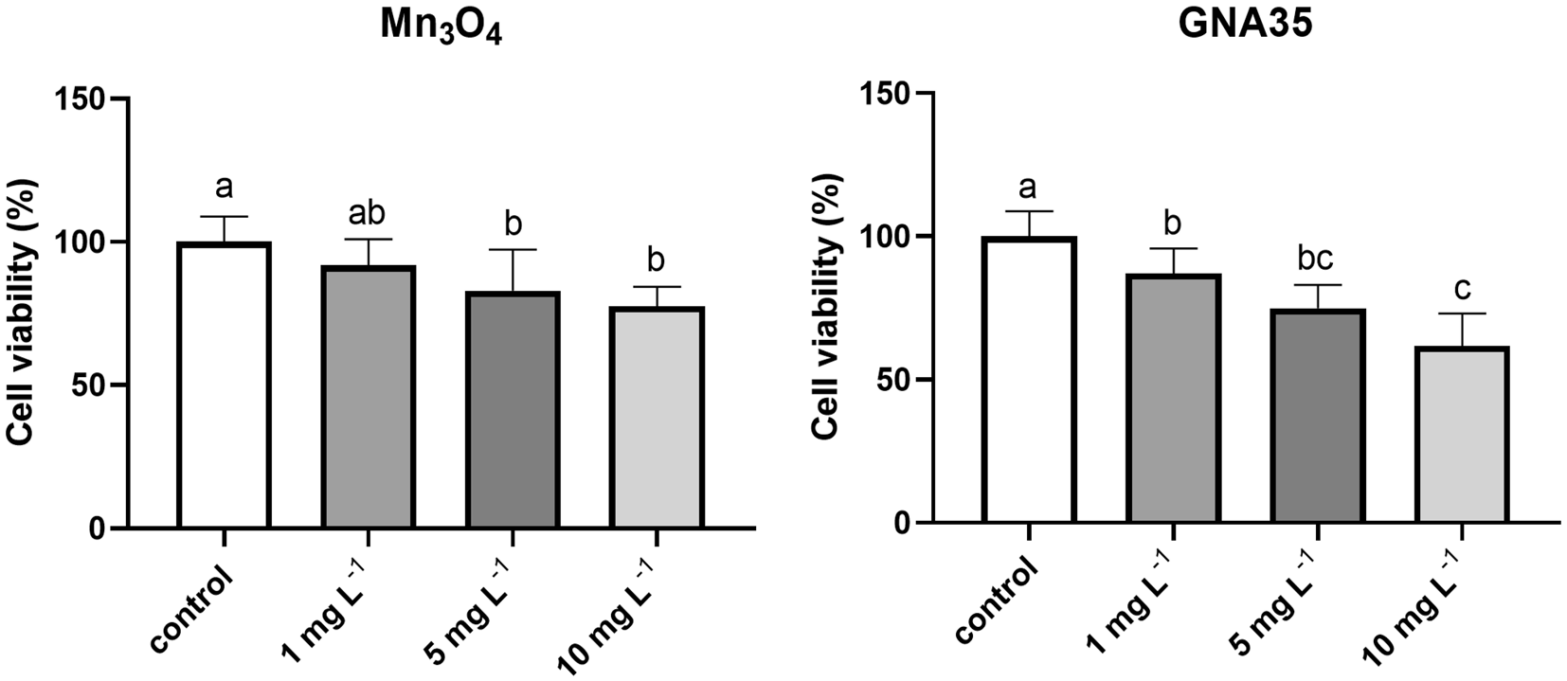
Viability of A549 cells exposed to different concentrations of Mn_3_O_4_ (left) and GNA35 (right) NMs. Results are expressed as % of control (non-exposed cells). Data represents the mean (± standard deviation, SD) of two independent experiments (6 biological replicates). Differences were established using a one-way ANOVA followed by multiple comparisons test (Tukey test) and considered significant when *P* ≤ 0.05. Different letters indicate significant differences between treatments.

The potential negative effect produced by the exposure of lung cells to the manganese oxide NMs under study was assessed employing alveolar carcinoma epithelial cells (A549) as model. First, the effects of the exposure to Mn_3_O_4_ and GNA35 were evaluated performing MTT viability test on A549 cell line. Prelimary experiments were carried out to evaluate the possible interference of the NMs with the MTT reagent or with the insoluble MTT-formazan crystal, none of the NMs increased the absorbance values as compared to control, suggesting that no unspecific interactions with the MTT reagent or with the MTT-formazan crystal can be observed for the studied NMs (see Supplementary Fig. S3). In the MTT assays the cells were exposed to different concentrations (1, 5 and 10 mg L^−1^) of both NMs for 24 h. The obtained results indicate that Mn_3_O_4_ and GNA35 are able to induce cytotoxic effects on the A549 cells at the selected concentrations, in a similar way, and in a concentration dependent manner (Fig. 4). Cells incubated in the presence of Mn_3_O_4_ nanoparticles at 5 mg L^−1^ and 10 mg L^−1^ present a statistically significant reduction (*P* ≤ 0.05) in viability when compared to the non-exposed cells condition (control). The response of A549 cells exposed to GNA35 nanoparticles was similar, where a statistically significant reduction in viability could be observed in cells exposed to 1 mg L^−1^ (*P* ≤ 0.05), 5 mg L^−1^ (*P* ≤ 0.0001), and 10 mg L^−1^ (*P* ≤ 0.0001).

**Figure 5:**
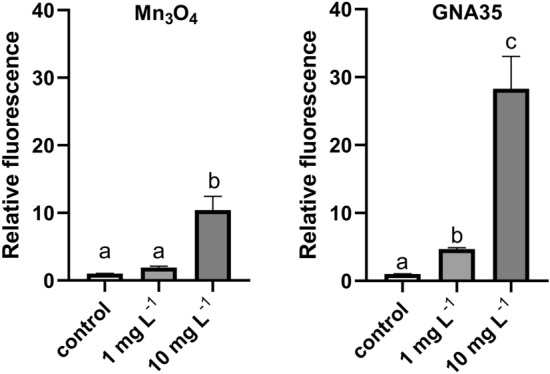
ROS production of A549 cells exposed to two concentrations of Mn_3_O_4_ (**left**) and GNA35 (**right**) NMs for 60 min. Data represents the mean of two independent experiments (6 biological replicates; ± standard deviation, SD). Differences were established using a one-way ANOVA followed by multiple comparisons test (Tukey test) and considered significant when *P* ≤ 0.05. Different letters indicate significant differences between treatments.

The induction of ROS after A549 cells exposure to two Mn_3_O_4_ and GNA35 concentrations (1 and 10 mg L^−1^) was evaluated as well. Before to perform the ROS detection assays, preliminary experiments were carried out to determine the possible interference of the NMs with optical detection of DCF fluorescence and with the catalytic activity of the NMs solutions in terms of H_2_DCF-DA oxidation. Both NMs did not show to cause any interference with the detection of fluorescence DCF (see "Supplementary Methods" and Supplementary Fig. S4). The DCFH-DA assay was employed to determine the levels of ROS at three exposure times (0, 30 and 60 min) (see Supplementary Fig. S6), by measuring the increase of relative fluorescence. As displayed in Supplementary Fig. S6, ROS formation induced by both NMs could be observed, in function of time and of the concentration employed. Differences in ROS levels amongst the distinct studied conditions were more evident after 60 min of exposure (Fig. 5). Both NMs induced ROS at 1 mg L^−1^, being this increase statistically significant in the case of GNA35. In addition, when the cells were exposed to 10 mg L^−1^ of Mn_3_O_4_ or GNA35 NMs the production of ROS was higher, showing statistically significant differences in comparison with the control, in all cases. We comparing the ROS produced by both NMs, it could be observed that GNA35 was able to induce 2 to 3 times higher ROS levels than Mn_3_O_4_ and the positive control (cells exposed to H_2_O_2_ (20 mM), see Supplementary Fig. S5) in A549 cells.

### Evaluation of GNA35 and Mn_3_O_4_ toxicity in the human colon cancer cell line HT29

**Figure 6:**
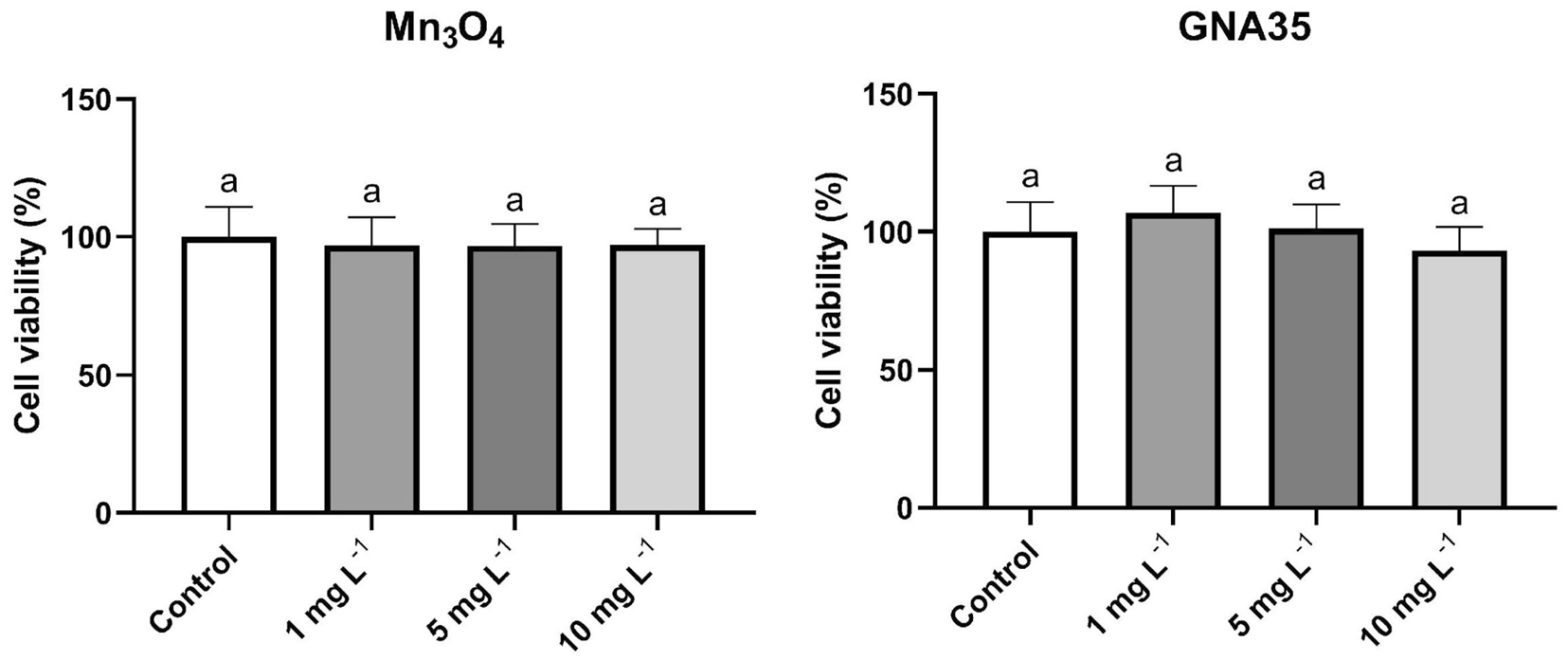
Viability of HT29 cells after exposure to Mn_3_O_4_ (left) and GNA35 (right) NMs. Results are expressed as % of control (non-exposed cells). Data represents the mean (± standard deviation, SD) of two independent experiments (6 biological replicates). Differences were established using a one-way ANOVA followed by multiple comparisons test (Tukey test) and considered significant when *P* ≤ 0.05. The same letter indicates no significant differences between treatments.

The colon cancer cell line HT29 was selected as a model to predict the potential toxicity induced by manganese oxide NMs upon exposure of human intestinal cells. The viability of HT29 cells, when exposed to Mn_3_O_4_ and GNA35 for 24 h, at 1, 5 and 10 mg L^-1^, was also determined by MTT assay. In contrast to the observations made when A549 cells were exposed to the same concentrations of the selected NMs, HT29 cells did not present significant cell viability differences with the control condition, at any concentration tested (Fig. 6).

**Figure 7:**
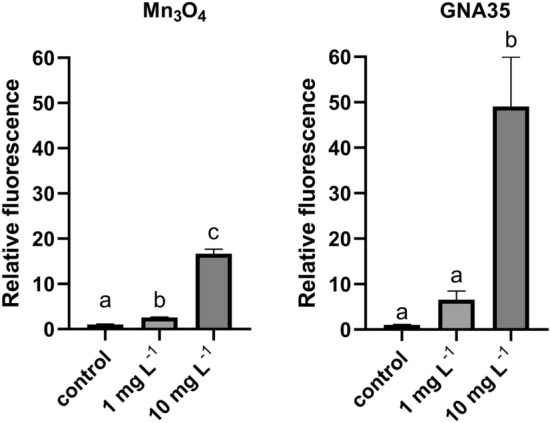
ROS production of HT29 cells exposed to two concentrations of Mn_3_O_4_ (**left**) and GNA35 (**right**) NMs for 60 min. Data represents the mean of two independent experiments (6 biological replicates; ± standard deviation, SD). Differences were established using a one-way ANOVA followed by multiple comparisons test (Tukey test) and considered significant when *P* ≤ 0.05. Different letters indicate significant differences between treatments.

The induction of ROS in HT29 cells by Mn_3_O_4_ and GNA35 was also assessed using the DCFH-DA assay, employing the same exposure conditions as those described for the A549 cell line. Both NMs provoked an increase in HT29 cells of ROS, in a similar fashion to that induced in A549 cells: the increase in ROS levels was directly associated to the increase of concentrations and the exposure time (see Supplementary Fig. S7). Again, ROS levels determined in the exposure conditions tested were highest after 60 min (Fig. 7). As observed in A549 cells, 1 mg L^−1^ of both NMs already increased the average ROS levels in HT29 cells, being only statistically significant in the Mn_3_O_4_ condition. ROS levels displayed by HT29 cells exposed to 10 mg L^−1^ of both NMs were also remarkably higher taking into account the levels of ROS presented by the positive control (cells exposed to H_2_O_2_ (20 mM), see Supplementary Fig. S5), particularly in case of GNA35, showing in all cases statistically significant differences in comparison with the non-exposed cells.

### Reconstructed human epidermis (RhE) response to GNA35 and Mn_3_O_4_ exposure

#### In Vitro EpiDerm skin irritation test

**Figure 8:**
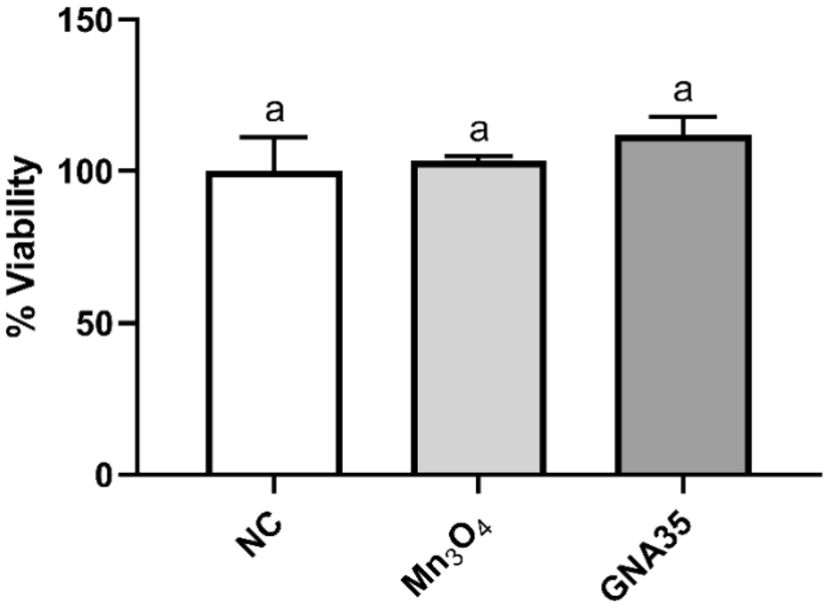
EpiDerm tissues were exposed to 500 mg L^−1^ of Mn_3_O_4_ and GNA35 nanomaterials for 1 h. Their viability was analysed by MTT assay, which is expressed as a percent of the values obtained for the non-exposed tissue (NC). Data represented the mean ± standard deviation (SD). Differences were established using a one-way ANOVA followed by multiple comparisons test (Tukey test) and considered significant when *P* ≤ 0.05. The same letter indicates no significant differences between treatments.

The potential of Mn_3_O_4_ and GNA35 to induce skin irritation was evaluated using the in vitro EpiDerm Skin Irritation Test (EPI-200-SIT), after confirming the inability of the NMs to interfere with and/or to reduce the MTT substrate, following OECD guidelines (Test N° 439). As described in the corresponding “Materials and Methods” section, the procedure involves the use of RhE, which consists of a 3D cell culture closely mimicking the biochemical and physiological properties of the upper parts of the human skin, employing the MTT assay to determine cell viability. According to EU and Globally Harmonized System of Classification and Labelling Chemicals, GHS, (R38/ Category 2 or no label), an irritant is predicted if the mean relative tissue viability of three individual tissues exposed to the test substance (in this case, the NMs under study) is reduced below 50% of the mean viability of the negative controls (tissues treated with PBS). A high concentration (500 mg L^−1^) of Mn_3_O_4_ and GNA35 did not reduce the viability of RhE (Fig. 8), while the positive irritation control (tissues exposed to 5% SDS) indicated by OECD TG439 reduced the RhE viability as expected (> 95%). The percentages of tissues viability are represented in the Supplementary Table S1. According to the obtained results, highly concentrated suspensions of both NMs (500 mg L^−1^) can be considered as non-irritant to the skin in the conditions tested.

In addition, in accordance with the criteria of OECD TG439 and following the manufacturer’s recommendations of the In Vitro Epiderm Skin Irritation Test (EPI-200-SIT), the evaluation of the cytokines release is an optional step to determine the potential irritant of a chemical. As suggest the In Vitro Epiderm Skin Irritation protocol, the concentration of IL-1α released from the RhE tissues into the assay medium after the exposure to Mn_3_O_4_ and GNA35 was also determined, by enzyme-linked immunosorbent assay (ELISA), and expressed as pg mL^−1^ released by RhE after each skin irritation test treatment. The IL-1α values obtained were 58.6 ± 34.0 pg mL^-1^ in case of the non-exposed tissues, 146.6 ± 79.9 pg mL^−1^ when the tissues were exposed to Mn_3_O_4_, and 69.7 ± 33.3 pg mL^−1^ when the tissues were exposed to GNA35. Although the average IL-1α released in the presence Mn_3_O_4_ was higher than in the rest of the conditions tested, the observed differences were not considered statistically significant.

## Discussion

The production and the use of Mn oxide NMs has increased in the last decades, leading to a major potential exposure and, consequently to a major risk for humans and the environment^16^. To address this issue is essential to gain knowledge in the potential toxicological effects that these NMs that may cause. In this context, the present work is focused on the comparative physico-chemical and toxicological evaluation of two Mn oxide nanomaterials, namely Mn_3_O_4_ and GNA35.

GNA35 was designed to improve the electrochemical functionalities of the commercial precursor Mn_3_O_4_, a necessary step for its use in applications such as the development of supercapacitators. The analysis of the physico-chemical properties of both NMs revealed similarities in terms of morphology and size, but differences in terms of stability, composition and electrochemical performance were observed. TEM analysis showed that both materials are nanoparticles with round shape and a similar diameter. In both cases, DLS and ζ-potential analysis revealed a certain colloidal instability when the NMs were resuspended in ultrapure water, higher in case of Mn_3_O_4_. Stability differences between GNA35 and Mn_3_O_4_ were more noticeable when the NMs were resuspended in human cells culture media. In this case, GNA35 maintained a similar stability to that observed in ultrapure water, while Mn_3_O_4_ instability prevented to perform accurate DLS and ζ-potential measurements. XRD analysis showed compositional differences between both NMs: Mn_3_O_4_ and GNA35 conserved the characteristic pattern of hausmannite, although the former showed to have a higher degree of impurities.

Surface area and cyclic voltammetry analyses were performed to understand the potential of the novel NM for its use in electrochemical applications. As expected, GNA35 showed improved properties with respect to the precursor NM. In terms of surface area, which was measured by analysing nitrogen adsorption–desorption isotherms, GNA35 (39 m^2^ g^−1^) showed to have a higher estimated BET area than Mn_3_O_4_ (14 m^2^ g^−1^). Cyclic voltammetry analysis allowed the calculation of the specific capacitance of the NMs, from galvanostatic charge/discharge measurements at 1 A g^−1^. The calculated specific capacitance for GNA35 (150 F g^−1^) was around one order of magnitude higher than that observed for Mn_3_O_4_ (16 F g^−1^). The obtained results demonstrated that GNA35 have significantly improved properties for charge accumulation when compared with the starting material, ensuring a better performance as electrode in an energy storage device.

Once the relevant physico-chemical properties of both NMs were understood, the toxicity of GNA35 and Mn_3_O_4_ was evaluated in different cellular models mimicking human tissues that could be directly exposed to them. There are numerous routes through which a chemical can enter the human body when considering occupational or environmental exposures, including, inhalation, ingestion, injection or contact with the skin^17^. The way of entry is a very important factor, and for this reason, we have evaluated the potential toxic effects of both NMs over three different human in vitro models suitable to perform a hazard assessment associated to potential inhalation, intestinal absorption and dermal contact: two cell lines of lung and gastrointestinal system, and an in vitro skin model.

As mentioned previously, the respiratory system is the main pathway for airborne contaminants entry into the body, allowing them to be transported to the other organs^18^. Considering this, we have analysed the cytotoxicity of Mn_3_O_4_ and GNA35 nanoparticles in the A549 cells. The obtained results (viability determination and ROS formation) showed a dose-dependent cytotoxicity within the employed concentration range (1–10 mg L^−1^). These results are in accordance with the work of Shaik et al. who showed as well a decrease in cell viability on the range of concentrations over 10–25 mg L^−1^ in response to Mn_3_O_4_ nanoparticles exposure^19^. Interestingly, manganese oxide NMs coated with polyethylene glycol (PEG) have shown very low toxicity, in terms of cell viability reduction, towards the same cell line, in a higher concentration range (200–1000 mg L^−1^)^20^. In regard to ROS induction, it is well known that metal oxide particles, including manganese oxide, induce ROS production, leading to cell damage^21^. Specifically, the generation of particularly high ROS levels upon exposure to Mn_3_O_4_ has been reported^22^, which is in concordance with the results obtained in the present study.

Other relevant via of potential exposure to nanoparticles is through intestinal absorption. Their accumulation in the intestine can produce toxicity and, consequently, increase the risk of developing colon cancer or other carcinomas^18^. In this context, we have studied the effect of Mn_3_O_4_ and GNA35 on the HT29 cells. In contrast to the results obtained for A549 cells, the viability of HT29 cells was not reduced after the exposure to both nanoparticles at 1, 5, 10 mg L^−1^. Khan et al. made a different observation in a previous study, where the exposure of HT29 cells to Mn_3_O_4_ reduced their viability at 5 and 10 mg L^−1^^23^. However, relevant characteristics that could affect the potential toxicity of the employed NMs, such as size, colloidal stability or BET were not revealed, which makes difficult to compare the obtained results in both research works. In any case, Mn_3_O_4_ and GNA35 enhanced ROS levels of HT29 cells in a similar way to what was observed for A549 cells, which shows their potential toxicity towards this colon cell line. In this regard, Choi et al. have demonstrated that manganese oxide nanoparticles are able to induce the production of ROS in different human cell lines^12^, suggesting a general ability of these NMs to generate cellular damage in human cells.

Skin contact is another relevant potential nanomaterial exposure route. It is the largest organ in the body (1.5–2 m^2^ of surface area), and it plays a very important role as a protective barrier against environmental allergens, pathogens, chemicals, and harmful materials^24^. In particular, metallic nanoparticles can release ions that could cause sensitization and irritation after the exposure, but no data has been made available in this regard for manganese oxide NMs. For this reason, more transdermal toxicity studies are required to understand the adverse effects of metallic nanoparticles on the skin^25,26^. In the present work, we have studied the irritant potential of Mn_3_O_4_ and GNA35 nanoparticles, in the form of highly concentrated liquid suspensions (500 mg L^−1^), on the reconstructed human epidermal model EpiDerm. Following the OECD Test N° 439 to determine in vitro skin irritation on RhE, we have observed that none of the NMs produce damage in the tissue. According to EU and Globally Harmonized System of Classification and Labelling Chemicals, GHS, (R38/Category 2 or no label), an irritant is predicted if the mean relative tissue viability of three individual tissues exposed to the test substance is reduced below 50% of the mean viability of the negative controls. In case of Mn_3_O_4_ and GNA35 nanoparticles, they did not reduce at all the viability of the in vitro model, so both nanoparticles can be considered as non-irritant in the studied conditions. Regarding the possible induction of an inflammatory skin process by Mn_3_O_4_ and GNA35, we evaluated the IL-1α secretion of the exposed RhE. The obtained results showed that none of the NMs produce a significant increase in the release of the IL-1α. So far, to our knowledge, there are no studies in the literature regarding the irritant or inflammatory potential of manganese oxide nanoparticles in RhE models. However, our results are in the same line with those described for other metallic nanoparticles (Fe NPs, Al NPs, Ti NPs and Ag NPs), which resulted to be non-irritant and non-inflammatory when the same test method and in vitro skin model was employed^27–30^.

Overall, the obtained results indicate that both the precursor product (Mn_3_O_4_) and the new synthesized nanomaterial with enhanced properties for its use in energy storage applications (GNA35) have similar hazard characteristics, inducing cytotoxicity effects in the selected lung and colon in vitro models, while lacking irritation potential in the skin model. In terms of the cytotoxicity effects observed, GNA35 induced the production of higher ROS levels than Mn_3_O_4_ in the A549 and HT29 human cell lines, which might be related to the higher specific surface and surface charge observed in GNA35. Both surface-related modes of action have been defined as key factors for nanomaterials toxicity ^31–33^.

## Conclusion

The results presented in this research article reveal the physico-chemical properties and the potential hazard of novel manganese oxide NMs (GNA35) developed for their use in the manufacture of energy storage devices. While GNA35 physico-chemical characteristics showed an enhanced surface area and electrochemical response in comparison with the precursor material employed for their synthesis (Mn_3_O_4_), the comparative study of their toxicological properties towards human in vitro models representing three potential exposure routes (inhalation, intestinal absorption and dermal contact) revealed a similar response in the selected exposure conditions. The NMs under study were able to reduce A549 cells viability, while no effect in the HT29 cells viability was observed. Both NMs provoked ROS generation in the lung and colon in vitro models. In both cases, GNA35 induced the production of higher ROS levels than Mn_3_O_4_, which might be related to the higher specific surface and surface charge observed in GNA35. In case of the dermal exposure, no effects were observed in the irritation and inflammation tests performed. Altogether, the presented results provide a new source of information related to the potential adverse effects of novel nanomaterials with enhanced electrochemical properties, with relevance for the implementation of safe-by-design strategies during their production and handling.

## Materials and methods

### Cell lines

The human alveolar carcinoma epithelial cell line A549 (ATCC, CCL-185) and human colorectal adenocarcinoma cell line HT29 (ATCC, HTB-38) were purchased from Sigma, and they were utilized for toxicological evaluation. A549 cells were cultured in Dulbecco’s Modified Eagle’s Medium (DMEM) supplemented with 10% fetal bovine serum (FBS) and 1% penicillin–streptomycin solution. HT29 cells were cultured in McCoy's 5A medium supplemented with 10% fetal bovine serum, 1% penicillin–streptomycin solution and 1% glutamine. Both cell lines were maintained at 37 °C in a humidified atmosphere containing 5% CO_2_.

The reconstructed human epidermal model EpiDerm was purchased from MatTek Life Sciences (MA, USA). The tissues were kept following the manufactures instructions (MatTek In Vitro Life Science Laboratories, 2020).

### Synthesis and characterization of the manganese oxide nanoparticles

GNA35 (Mn_3_O_4_ nanoparticles) was synthetized through a novel nanotechnological process based on patented procedures (Patent number ES2678419A1), using commercial Mn_3_O_4_ as precursor (Strem Chemicals, MA, USA). DLS and the ζ-potential determination was done using a Zetasizer Nano ZS90 (Malvern Instruments). The nanomaterials suspensions (20 μg mL^−1^) were sonicated for 10 min prior to the analysis. The hydrodynamic diameter was determined by Dynamic Light Scattering (DLS), for this analysis the samples were diluted 10 times (1:10). ζ-potential was measured using the M3-PALS method. The TEM analysis was performed using a JEOL 1011 high-resolution (HR) TEM. Samples were deposited on standard copper grids. The chemical composition and crystalline phase of the nanoparticles were determined by X-ray diffraction analysis, using X-Ray Polycrystalline Diffraction technique. The X-Ray power diffraction was recorded on a Panalytical XPERT Pro system equipped with Cu K radiation at wavelength (λ) 0.15418 nm. BET analysis was performed by determining the porosity of both nanomaterials, analyzing their nitrogen adsorption–desorption isotherms, employing a Micromeritics ASAP 2420 V2.09 equipment. An Arbin (BT-G-5-10A) potentiostat was used for the electrochemical evaluation of the materials (Mn_3_O_4_ and GNA35). Cyclic voltammetry analyses were recorder at 5 mV s^−1^ in a potential range of − 0.8–0.3 V and − 0.6–0.4 V for Mn_3_O_4_ and GNA35, respectively. Galvanostatic charge–discharge analyses were recorded at 1 A g^−1^ to estimate the specific capacitance (F g^−1^) for each material. Fabrication of the electrodes was done mixing Mn_3_O_4_ or GNA35 (80%) with carbon black (10%) and poly(vinylidene fluoride) (10%) using an agate mortar, followed by the addition of ethanol to make a homogeneous paste. Nickel foam was used as current collector. The electrodes were dried at 90 °C overnight. The electrochemical measurements were carried out in a 3-electrode cell using previously manufactured electrodes as working electrodes, and platinum wire and Ag/AgCl as counter and reference electrodes, respectively. A solution of 1 M Potassium hydroxide (KOH) was employed as electrolyte.

### Toxicology assays

Nanomaterials stock suspensions for toxicology assays were prepared at 4 mg mL^−1^ in water (H_2_O_2_). Prior to the exposure assessment, single use aliquots were sonicated in a water bath for 20 min in order to facilitate their dissolution. In each experiment it was employed new sonicated and then they were discarded.

### MTT assay

Plates for 3-(4,5-dymethylthiazol-2-yl)-2,5-diphenyltetrazolium bromide (MTT) assays were seeded with 5 × 10^3^ cells per well (A549 cell line), or 1 × 10^4^ cells per well (HT29 cells) and left for incubation at 37 °C for 24 h at 5% CO_2_ environment. After the first stage of incubation, media was removed, and the cells were treated with different concentrations of NMs. The untreated cells were used as negative control and the cells exposed to water were employed as positive control. All solutions were prepared in the corresponding growth media but lowering the fetal bovine serum (FBS) to 1% (v/v) and without modification of the antibiotics. Exposure volume was always 200 µL (the volume of nanoparticles represents a 2% of the total volume of culture medium added to each well). Incubation lasted for 24 h at 37 °C, 5% CO_2_ environment. After 24 h exposure, media with nanomaterials were retrieved and wells washed with sterile Dulbecco`s Phosphate—Buffered Saline (DPBS). A hundred µL of growth media supplemented with 0.5 mg mL^-1^ MTT were added per well, and after 3 h incubation it was removed. The resulting formazan crystals were solubilized with 100 µL dimethyl sulfoxide (DMSO), incubated for 15 min. at 37 °C in darkness, under continuously shaking. Absorbance was measured at 590 nm with a microplate reader (BioTek Synergy HT). Three replicates per dose were included in each experiment and at least two independent experiments were performed.

Particle interference control experiments were performed following the protocol adapted from Chng et al.^34^ before to perform the MTT assays to ensure that the cytotoxicity results obtained are not affected by the interference of the Mn_3_O_4_ and GNA35 NMs with the MTT reagent or with the insoluble MTT-formazan crystal (see "Supplementary Methods" and Supplementary Fig. S3).

### ROS detection assay

We followed a reactive oxygen species (ROS) assay method adapted from Domi et al.^35^, using a quantitative method for measuring ROS upon exposure to nanomaterials. In brief, A549 and HT29 cells were seeded in 96 micro-well plates, at a density of 3 × 10^4^ cells per well. After 24 h incubation, growth media was removed and washed once with 1 × Hank’s Balanced Salt Solution (HBSS) without phenol red pH 7.2 (NaOH adjusted), then incubated with 50 µM 2,7-dichlorofluorescein diacetate (DCFH-DA) for 30 min at 37 °C in darkness, with the exception of three wells incubated only with HBSS without dye. Upon incubation, we removed the buffer with dye and washed cells once with HBSS. Previously prepared nanomaterials dissolved in HBSS were added to each well at different concentrations ranging from 1 to 10 mg L^−1^, while cells incubated with HBSS alone were employed as negative control and cells exposed to H_2_O_2_ (20 mM) were used as positive control (see Supplementary Fig. S5). Exposure volume was always 200 µL (the volume of nanoparticles represents a 2% of the total volume of culture medium added to each well). We measured fluorescence in a microplate reader (BioTek Synergy HT, excitation wavelength, 485/20; emission wavelength 520/20) after 0, 30, and 60 min of exposure. Three biological replicates were included in each independent assay.

In order to predict the compatibility of the NMs with the optical detection of DCF fluorescence, interference tests were carried out following the adapted protocols describe by Vrček et al.^36^ and Kroll et al.^37^ (see "Supplementary Methods" and Supplementary Fig. S4).

### In Vitro EpiDerm skin irritation test

The skin irritation test was performed according to the in vitro EpiDerm Skin Irritation Test (EPI-200-SIT) protocol (MatTek In Vitro Life Science Laboratories, 2020). The reconstructed human epidermal model EpiDerm (EPI-200-SIT, MatTek, Bratislava) consists of normal human-derived epidermal keratinocytes cultured to form a multilayered differentiated model of the human epidermis. Upon receipt, the tissues were inspected for damage according to manufacture instructions, transferred to 6-well plates prefilled with 0.9 mL of assay medium (EPI-100-NMM provided with EpiDerm tissues) and incubated at optimal conditions (37 °C ± 1 °C, 5 ± 1% CO_2_, 90 ± 10% relative humidity (RH)) for 1 h. Then, the tissues were transferred to fresh media and incubated overnight (18 ± 2 h) at optimal conditions to release transport-stress. Afterwards, the tissues were exposed to the NMs (25 µL) during 60 min (37 °C  ± 1 °C, 5 ± 1% CO_2_, 90 ± 10% RH). Three tissues were used per test material, as well as for the positive and negative controls. Tissues exposed to DPBS were used as negative control, while tissues exposed to a 5% solution of sodium dodecyl sulfate (SDS) were used as positive control. After exposure, tissues were rinsed with DPBS (15 times), blotted in a sterile blotting paper, and dried with a sterile cotton-tipped swab to remove the test substances from the surface of the tissue, subsequently transferred to a 6-well plate with 0.9 mL culture medium, and incubated at optimal conditions for 24 ± 2 h. Finally, the medium was collected for analysis of cytokines and the tissues were incubated again, in fresh medium, at optimal conditions for 18 ± 2 h.

Tissues viability after the RhE exposure to the nanomaterials was determined using the MTT viability assay, following procedures described in the OECD guideline Test N° 439. At the end of the 18 ± 2 h of incubation, the tissues were placed in a 24-well plate containing 0.3 mL per well of the MTT (1 mg mL^−1^) and incubated for 3 h at optimal conditions. After this step, tissues were rinsed twice with PBS, and formazan crystals were solubilized adding 2 mL per tissue of isopropanol (MTT-100-EXT, included in the MTT-100 kit, MatTek, Bratislava). The plates were set on an orbital shaker for 2 h at room temperature. At the end of the extraction period, tissues were pierced with an injection needle and the extract ran into the well from which the insert was taken. Afterwards, the inserts were discarded, and the extraction solutions were homogenized and transferred to a 96-well plate. Tissue viability is reported as % of negative control, measuring the OD of each isopropanol extract in duplicate at 570 nm by using a plate reader (BioTek Synergy HT). Isopropanol alone was used as a blank. The percent viability of each tissue was calculated relative to negative control using the following equation:

$$\% {\text{ Viability}}_{{{\text{tissue}}}} \, = \,\left[ {{\text{OD}}_{{{\text{tissue}}}} /{\text{Mean OD}}_{{{\text{NC}}}} } \right]\, \times \,{1}00\% .$$

The inability of the NMs to interfere with and/or to reduce the MTT was determined following the guideline recommendations.

### Cytokine release assay

After 24 h post-treatment with Mn_3_O_4_, and GNA35 nanoparticles, culture media from the RhE tissues was recollected and stored at – 20 °C. The concentration of IL-1α was evaluated by ELISA test (Diaclone), following the manufacturer’s instructions. Results are expressed as pg mL^−1^ of cytokines released in the media.

### Statistical analysis

The results are represented as mean ± SD. Differences between the different treatments were established using a one-way ANOVA followed by multiple comparisons test (Tukey test). Statistical test was carried out using GraphPad Prism Software, Inc (version 8.0.2). Statistical significance was considered at *P* ≤ 0.05.

## Supplementary Information

Supplementary Information.

## Data Availability

The datasets generated and analysed during the current study are available from the corresponding author on reasonable request.
