# Supplementary material for: Toxicology assessment of manganese oxide nanomaterials with enhanced electrochemical properties using human in vitro models representing different exposure routes
Source: Sci Rep. 2022 Dec 5;12:20991. doi: 10.1038/s41598-022-25483-w (PMC9723098; doi:10.1038/s41598-022-25483-w)
Supplement: Supplementary file 1 — Supplementary Information. [file 41598_2022_25483_MOESM1_ESM.docx]

**Supplementary file**

**Toxicology assessment of manganese oxide nanomaterials with enhanced electrochemical properties using human *in vitro* models representing different exposure routes**

Natalia Fernández-Pampín^1*^, Juan José González Plaza^1*^, Alejandra García-Gómez^2^, Elisa Peña^2^, Carlos Rumbo^1^, Rocío Barros^1^, Sonia Martel-Martín^1^, Santiago Aparicio^1,3^, Juan Antonio Tamayo-Ramos^1†^

1 International Research Center in Critical Raw Materials for Advanced Industrial Technologies (ICCRAM), Universidad de Burgos, 09001 Burgos, Spain.

2 Gnanomat S.L., Campus Cantoblanco, Madrid Science Park, c/ Faraday 7, 28049, Madrid, Spain

3 Department of Chemistry, Universidad de Burgos, 09001, Burgos, Spain.

*Equal contribution

†Author to whom correspondence may be addressed: Juan Antonio Tamayo Ramos; jatramos@ubu.es; ja.tamayoramos@gmail.com


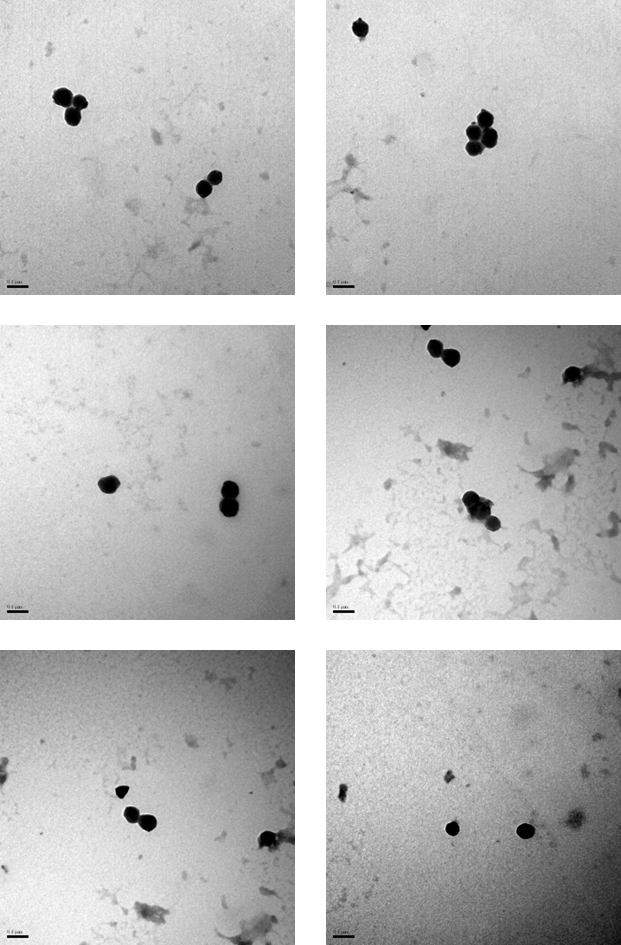


Figure S1. TEM images of the precursor material Mn_3_O_4_. The displayed scale bar indicates 100 nm.

_
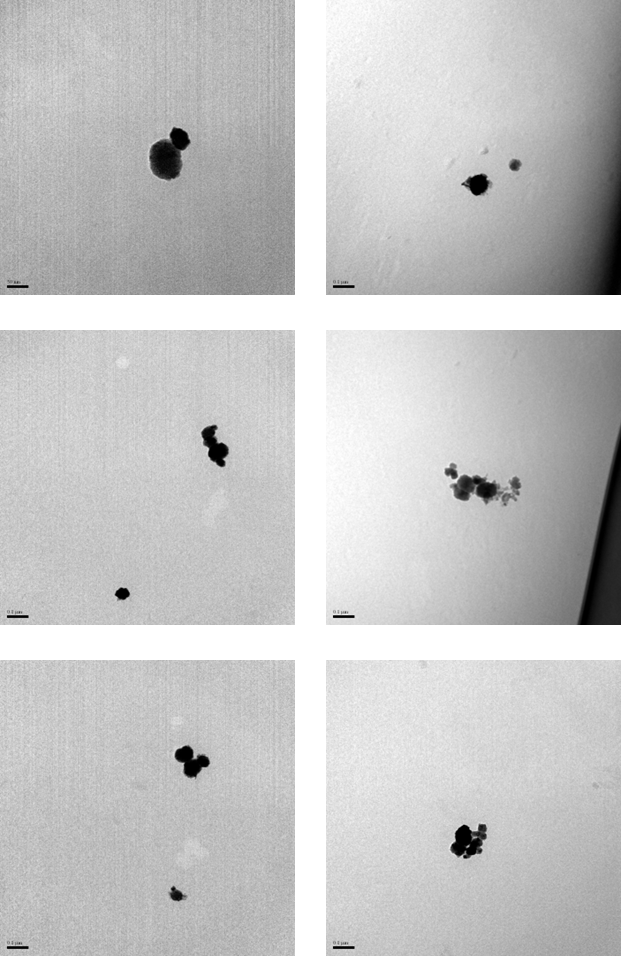
_

Figure S2. TEM images of the synthesized material GNA35. The displayed scale bar indicates 100 nm.

**Methods**

The interference of Mn_3_O_4_ and GNA35 nanomaterials with the MTT to produce formazan was determined under cell-free conditions. The nanomaterial solutions (1, 5 and 10 mg L^-1^) were mixed with MTT (0.5 mg mL^-1^) at a radio 1:1 and incubated at 37 ºC for 3 h. Then, DMSO was added at a ratio 2:1 to the MTT-nanomaterial solutions mixture and incubated for 10 minutes at 37 ºC. The final mixture was centrifuged at 5000 × g for 10 minutes, and the supernatant absorbance was measure at 570 nm in a microplate reader (BioTek Synergy HT). Four replicates were included in the assay. None of the nanomaterials reacted with the MTT reagent in the studied conditions (Fig. S3a).

In addition, we determine in absence of cells the degree of adsorption of the insoluble formazan crystals to the NMs solutions by the incubation of Mn_3_O_4_ and GNA35 solutions with MTT for 3 h at 37 ºC. After that, the MTT was reduced to formazan by using ascorbic acid, 0.16 mL of the MTT-NM solutions mixture were incubated with 0.066 mL of ascorbic acid (0.05 mM) for 60 minutes at 37 ºC. Then, DMSO was added to MTT- NM solutions-ascorbic acid mixture at a ratio 2:1 and it was incubated for 10 minutes at 37 ºC. The final mixture was centrifuged at 7000 × g for 5 minutes. Finally, the supernatant absorbance was measured at 570 nm with a microplate reader (BioTek Synergy HT). Four replicates were included in the assay. None of the NMs caused a decrease in the measured formazan concentration, suggesting that there was not adsorption of the NMs to the insoluble MTT-formazan crystals (Fig. S3b).


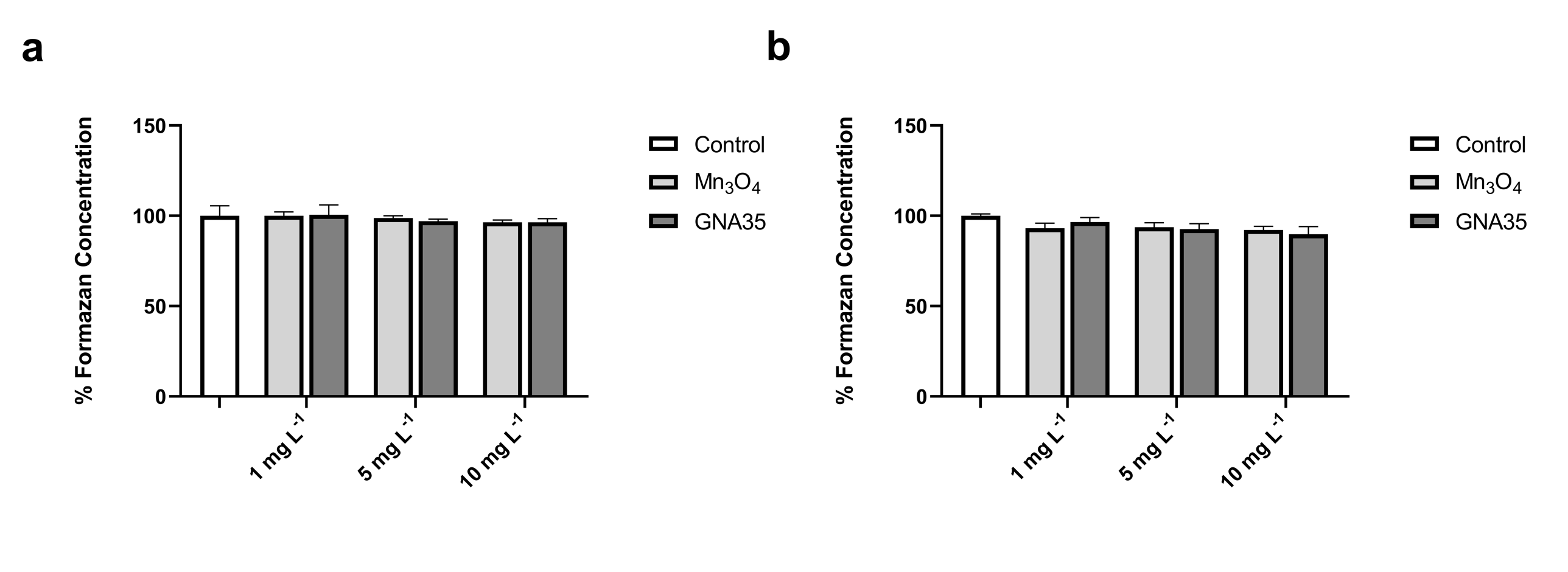


**Figure S3.** Concentration (%) of formazan produced by Mn_3_O_4_ and GNA35 under cell-free conditions. a) Effect of NM solutions on the generation of formazan through the reduction of MTT reagent. b) Acid ascorbic was added to the NMs solutions to reduce the remaining soluble MTT to formazan. Data represent the mean of 4 replicates (± standard deviation, SD).

The interference of the NMs with optical detection of DCF fluorescence was determined under cell-free conditions and using A549 cells by replacing the assay substrate H_2_DCF-DA by DCF (50 µM). In the cell-free experiments, the NMs solutions (1 and 10 mg L^-1^) were incubated with DFC (50 µM) for 60 minutes at 37 ºC. After that, the fluorescence was measured in a microplate reader (BioTek Synergy HT, excitation wavelength, 485/20; emission wavelength 520/20). Four replicates were included in the assay. All the NMs did not show to cause any interference with the detection of fluorescence DCF at concentrations of 1 and 10 mg L^-1^ (Fig. S4a).

In the cellular assays, cells monolayers were prepared as describe in the Materials and Methods section (ROS detection assay in A549 cells) and they were incubated with 100 µL NMs solutions at 1 and 10 mg L^-1^ for 60 minutes at 37 ºC. Then, the cells were washed with HBSS and treated with DCF (50 µM). Fluorescence was measured after 60 minutes in a microplate reader (BioTek Synergy HT, excitation wavelength, 485/20; emission wavelength 520/20). Four replicates were included in the assay. The obtained results indicated that Mn_3_O_4_ and GNA35 do not interfere in the detection of the DCF fluorescence at concentrations of 1 and 10 mg L^-1^ (Fig. S4b).


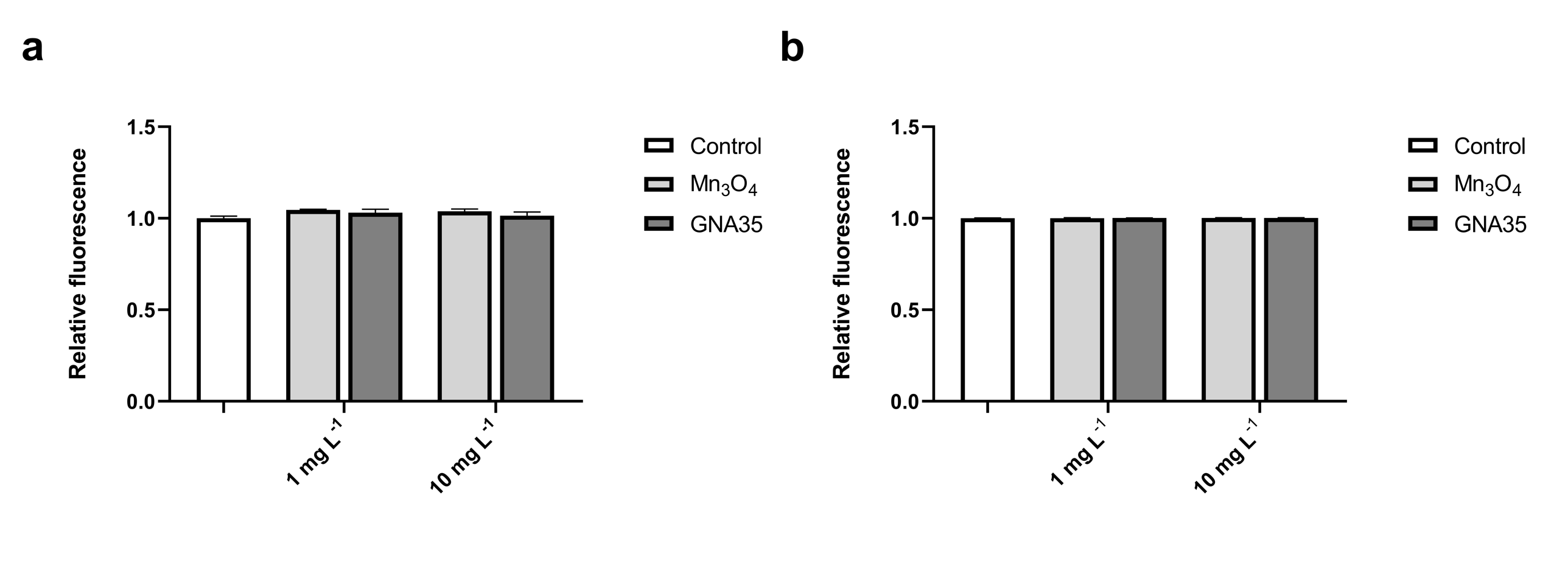


**Figure S4.** a) Interference of Mn_3_O_4_ and GNA35 with DCF fluorescence under cell-free conditions. b) Interference of Mn_3_O_4_ and GNA35 with DCF fluorescence using A549 cells. Results are expressed as the relative fluorescence value to the control, which was assigned a value of 1. Data represent the mean of 4 replicates (± standard deviation, SD).

Moreover, the catalytic activity of the NMs solutions in terms of H_2_DCF-DA oxidation was evaluated. The NMs solutions (1 and 10 mg L^-1^) were incubated with H_2_DCF-DA (50 µM) for 60 minutes at 37 ºC. After that, the fluorescence was measured in a microplate reader (BioTek Synergy HT, excitation wavelength, 485/20; emission wavelength 520/20). However, the levels of DCF fluorescence emitted by the NMs solutions were lower, showing that none of the NMs caused any effect on DCF fluorescence in the studied conditions.





**Figure S5.** A549 cells (a) and HT29 cells (b) treated with H_2_O_2_ 20 mM were used as internal positive controls in the ROS detection assays. The fluorescence was measured at 0, 30 and 60 minutes. Results are expressed as the relative fluorescence value to the control (untreated cells), which was assigned a value of 1. Data represent the mean of 3 replicates (± standard deviation, SD).


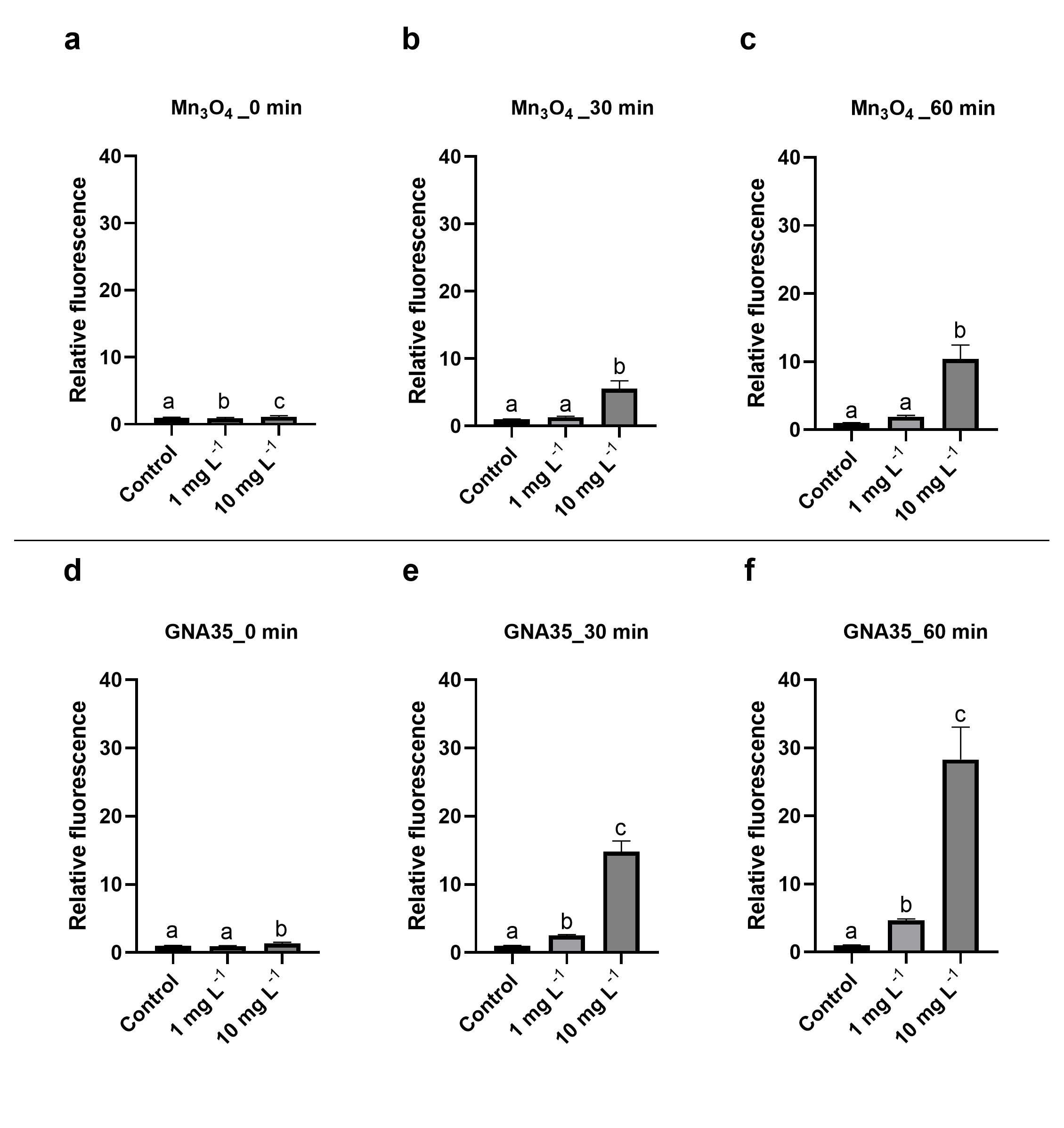


**Figure S6.** Comparison of the A549 ROS response against Mn_3_O_4_ (a-c) and GNA35 (d-f) nanoparticles at 0 minutes (a, d), 30 minutes (b, e) and 60 minutes (c, f). The charts have been represented at the same scale on the Y-axis. Data represent the mean of 2 independent experiments (± standard deviation, SD). Differences were established using a one-way ANOVA followed by multiple comparisons test (Tukey test) and considered significant when *P* ≤ 0.05. Same letters show no significant differences between treatments.


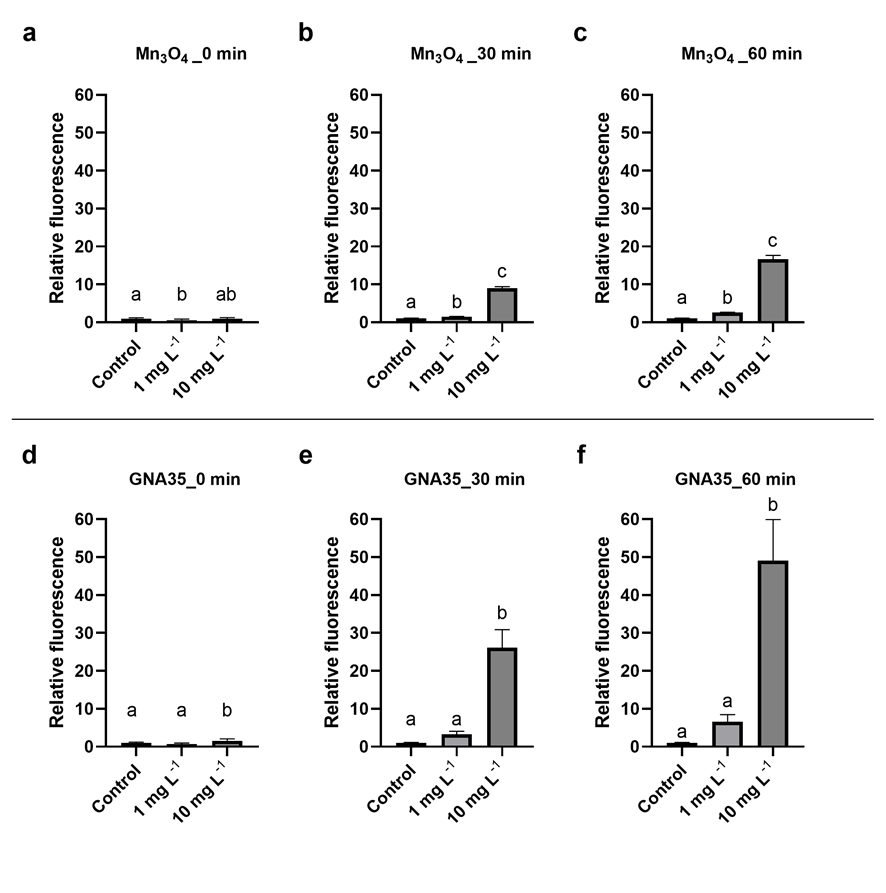


**Figure S7.** Comparison of the HT29 ROS response against Mn_3_O_4_ (a-c) and GNA35 (d-f) nanoparticles at 0 minutes (a, d), 30 minutes (b, e) and 60 minutes (c, f). The charts have been represented at the same scale on the Y axis. Data represent the mean of 2 independent experiments (± standard deviation, SD). Differences were established using a one-way ANOVA followed by multiple comparisons test (Tukey test) and considered significant when *P* ≤ 0.05. Same letters show no significant differences between treatments.

**Table S1.** Assessment of skin irritation properties of Mn_3_O_4_ and GNA35 using the *in vitro* EpiDerm Skin Irritation Test (EPI-200-SIT).

|  | Dose | % Viability (mean ± SD) | Viability reduction (%) | Irritancy classification |
| --- | --- | --- | --- | --- |
| NC | 0.99 µL mm^2^ (PBS 1X) | 100 ± 11 | 0 | NI |
| Mn_3_O_4_ | 0.99 µL mm^2^ (500 mg L^-1^) | 103.37 ± 1.47 | - 3.37 | NI |
| GNA35 | 0.99 µL mm^2^ (500 mg L^-1^) | 111.92 ± 5.90 | - 11.92 | NI |
